# Scaffold Application for Bone Regeneration with Stem Cells in Dentistry: Literature Review

**DOI:** 10.3390/cells13121065

**Published:** 2024-06-19

**Authors:** Elham Saberian, Andrej Jenča, Yaser Zafari, Andrej Jenča, Adriána Petrášová, Hadi Zare-Zardini, Janka Jenčová

**Affiliations:** 1 Klinika of Stomatology and Maxillofacial Surgery Akadémia Košice Bacikova, Pavol Jozef Šafárik University, 040 01 Kosice, Slovakia; 2 Lyles School of Civil Engineering, Purdue University, West Lafayette, IN 47907, USA; 3 Department of Biomedical Engineering, Meybod University, Meybod 89616-99557, Iran

**Keywords:** bone regeneration, stem cells, scaffold, tissue engineering, dental applications

## Abstract

Bone tissue injuries within oral and dental contexts often present considerable challenges because traditional treatments may not be able to fully restore lost or damaged bone tissue. Novel approaches involving stem cells and targeted 3D scaffolds have been investigated in the search for workable solutions. The use of scaffolds in stem cell-assisted bone regeneration is a crucial component of tissue engineering techniques designed to overcome the drawbacks of traditional bone grafts. This study provides a detailed review of scaffold applications for bone regeneration with stem cells in dentistry. This review focuses on scaffolds and stem cells while covering a broad range of studies explaining bone regeneration in dentistry through the presentation of studies conducted in this field. The role of different stem cells in regenerative medicine is covered in great detail in the reviewed literature. These studies have addressed a wide range of subjects, including the effects of platelet concentrates during dental surgery or specific combinations, such as human dental pulp stem cells with scaffolds for animal model bone regeneration, to promote bone regeneration in animal models. Noting developments, research works consider methods to improve vascularization and explore the use of 3D-printed scaffolds, secretome applications, mesenchymal stem cells, and biomaterials for oral bone tissue regeneration. This thorough assessment outlines possible developments within these crucial regenerative dentistry cycles and provides insights and suggestions for additional study. Furthermore, alternative creative methods for regenerating bone tissue include biophysical stimuli, mechanical stimulation, magnetic field therapy, laser therapy, nutritional supplements and diet, gene therapy, and biomimetic materials. These innovative approaches offer promising avenues for future research and development in the field of bone tissue regeneration in dentistry.

## 1. Background

A crucial component of orthopedic and dental care is bone regeneration, especially when it comes to spinal surgery, traumatic and congenital defects, and improving bone support around biomedical implants [1]. Despite the variety of techniques employed in clinical settings, such as bone grafting, guided bone regeneration, and distraction osteogenesis, achieving successful and functional bone regeneration remains a significant challenge [2]. Although autogenous bone transplants are considered the gold standard, their practical applicability is limited due to morbidity at the donor site and the scarcity of sufficient bone volume. Consequently, these limitations have propelled scientific attention to examining synthetic and xenograft biomaterials as possible scaffolds or alternatives to bone grafting. The human bone regeneration process mainly involves a variety of cellular and molecular interactions, such as inflammation, stem cell migration and differentiation into osteoblasts, synthesis of the bone matrix, and remodeling of the bone structure leading to the restoration of broken bone, preservation of its integrity, and guarantee of healthy skeletal function. The process of bone regeneration is crucial for the creation and restoration of bone tissue, particularly during the healing of fractures and injuries, and guarantees the integrity of the skeletal system [3]. A wide range of materials has been developed and evaluated regarding their potential for bone regeneration [4]. A common characteristic among these materials is their ability to create a three-dimensional (3D) cellular environment. Notably, two-dimensional (2D) and three-dimensional (3D) cells exert distinct behaviors that impact critical factors such as cellular mechanics, nutrient diffusion, and cell-to-cell communication [5]. Furthermore, for optimal functionality, synthetic implantable biomaterials meant to replace natural bone should mimic the structure and mechanical properties of bone [6]. Bone fillers and porous biomaterials are two key types of bone-regenerating materials [7]. Bone fillers, made from calcium-based materials, are easy to mold and place around fractures, sealing them and reducing bone tissue loss. However, they are brittle and unsuitable for endogenous mesenchymal stem cell (MSC) infiltration due to their lack of porosity and proper pore distribution. Porous biomaterials, categorized into ceramics, metals and alloys, and polymers, are favored for their ability to facilitate cellular infiltration and nutrient diffusion, and their porous structure mimics the natural three-dimensional environment, encouraging cell growth and interactions [8]. Stem cells from dental tissues, including pulp, periodontal ligament, and tooth germ, are being explored for their potential in dental regeneration [9]. By utilizing the body’s regenerative abilities, stem cell transplantation offers a cutting-edge method to transform conventional treatments [10] and possibly provide more individualized and effective solutions for a range of oral [11] and jaw [12] health issues. Recent studies in the field of regenerative medicine have indicated that mesenchymal stem cell-derived exosomes may represent a potent new therapeutic option [13]. Mesenchymal stem cells are multipotent stromal cells with the ability to self-renew and differentiate into many lineages. MSCs also release bioactive compounds with the potential to affect immune function and induce tissue regeneration. Consequently, MSCs are highly regarded as a promising tool for tissue regeneration [14]. Because of their noted functional decline and inhibited regenerative abilities, autologous MSCs derived from the patient’s own bone marrow or periodontal ligament have demonstrated limited success in treating osteoporosis and bone defects [15]. Allogeneic MSCs derived from healthy donors may show diminished potential for bone regeneration following extended in vitro cultures [16]. Although allogeneic MSCs have low immunogenicity and immunomodulatory features, they can elicit immunological responses and inflammation, which cause undesired bone regeneration outcomes. The recipient’s immune status during systemic infusion determines whether MSC transplantation will be successful in treating skeletal diseases [17]. Due to compromised immunomodulation, comorbidities like diabetes can reduce the effectiveness of infused MSCs in the treatment of osteoporosis [18]. Similarly, the ability of MSC-based bone tissue engineering to repair bone defects may be diminished by recipient conditions linked to estrogen-deficient osteoporosis [19]. It should be mentioned that high doses of MSCs can cause potentially fatal disseminated intravascular thrombosis and that MSCs may not be entirely compatible with the recipient’s circulation [20]. Although some studies indicate that bone marrow-derived MSCs are superior to adipose-derived MSCs, the use of adipose-derived MSCs for bone repair is still up for debate. In contrast, MSCs derived from umbilical cord matrix have shown comparable osteogenic potential to MSCs from bone marrow under two- and three-dimensional differentiation conditions [21]. Hence, the incorporation of MSCs from bone marrow and adipose tissue into scaffolds presents a promising opportunity for cartilage restoration [22]. In vitro instances of trilineage differentiation utilizing bone marrow, adipose tissue, and dental pulp-derived MSCs demonstrate their appropriateness for tissue engineering uses. MSCs found in dental pulp, including adult dental pulp stem cells (DPSCs) and stem cells derived from human exfoliated deciduous teeth (SHED), have garnered significant interest owing to their remarkable capacity for bone formation, favorable paracrine and immunomodulatory attributes, and rapid rate of proliferation [23]. Moreover, DPSCs and SHED from extracted and discarded teeth are easily isolated and accessible. They have an advantage over bone marrow and embryonic stem cells, offering a plentiful and low-risk source of cells for regenerative therapy [24]. Severe oral and maxillofacial bone defects pose significant challenges in terms of treatment. DPSCs may be suitable for regenerative therapy, but further understanding, optimization, and safety considerations are needed [25]. DPSCs are advantageous due to their ease of access, high extraction efficiency, and versatility. They can differentiate into osteoblasts and endotheliocytes, contribute to 3D woven bone tissue chips, and exhibit immune privileges and anti-inflammatory properties, making them a promising candidate for allogenic tissue grafts and clinical trials [26]. The scaffold, as a crucial element of tissue engineering, promotes regeneration by acting as a mechanical support network to hold recruited stem cells in place and to allow growth factors to attach. The integration of stem cells with scaffold materials and the recruitment of growth factors are critical factors that determine the extent of success in bone regeneration. Ongoing developments in MSC-based bone regeneration methods include cell-sheet/cell-aggregate, nanotechnology-enhanced scaffold materials, 3D-bioprinting, microencapsulation, and the use of cytokines that amplify the MSC function through preconditioning or co-delivery systems [27]. Even though MSC-based bone regeneration has made great strides, full and long-lasting bone healing still faces obstacles. Osteoporosis, diabetes, and estrogen deficiency are examples of pathological factors that may limit the effectiveness of current treatments that target endogenous MSCs and contribute to non-healing bone. Furthermore, prolonged use of non-specific drugs may also result in adverse effects, such as rapamycin’s suppression of immune function [28]. Polymeric scaffold materials have been explored for many clinical applications, such as maintenance of the alveolar ridge, enhancement of the maxillary sinus, reconstruction of temporomandibular joint TMJ, and endodontic regeneration [29]. Tissue engineering techniques have been used to boost the in vitro functionality of DPSCs, combining material and engineering techniques [30,31]. Several techniques, including hydrogel matrix support, scaffold reconstruction, and artificial patterning are used to develop tissue structures and functions. However, hydrogels are mainly accompanied by mechanical shortcomings, necessitating scaffolds for cellular behavior and function. The selected scaffold materials for use in regenerative medicine should be non-toxic with minimal side effects, high biocompatibility, and low immunogenicity [32]. Moreover, challenges include the loss of MSC potency after prolonged culture and the potential for senescence in autologous bone marrow aspirates, both integral parts of MSC application. Therefore, MSCs from other sources, including dental pulp, umbilical cord tissue, and adipose tissue, which are thought to have higher MSC concentrations, have been introduced [33].

This review represents the studies conducted on scaffold application for bone regeneration. We firstly represent the scaffold application in bone regeneration with stem cells. Secondly, the different types of scaffolds used, and their properties and effectiveness in promoting bone regeneration, are discussed. In the third step, we explore the contribution of stem cells to improve the regeneration process and scaffold integration in tissue engineering for bone regeneration. 

## 2. Scaffold Application in Bone Regeneration with Stem Cells

Scaffolds are an essential tissue engineering tool in stem cell-assisted bone regeneration, which offers structural support and a natural extracellular matrix-like environment for osteogenic cell contact and proliferation [34]. Bioactive molecules in scaffolds boost the MSCs’ osteogenic potential by invigorating precursor cells and bone metabolism [35]. Customized scaffold structures optimize cell seeding, growth, and vascularization. The success of using different organic and inorganic scaffold materials for bone regeneration in vitro and in vivo depends on the types of accompanying stem cells, and the scaffold’s capacity to provide an appropriate environment for stem cell development [36]. Agents that stimulate endogenous MSCs, such as SDF-1, CTGF, and TGFβ3, have been delivered using scaffold-based methods to improve bone regeneration in defect models [37]. Applications of tissue engineering for locoregional conditions have been successful, including periodontal structures, local osteoporosis, and defects of the long and craniofacial bones [38]. Bone regeneration using stem cells and scaffolds has been extensively researched. Studies have revealed that the combination of scaffold and human deciduous tooth exfoliated stem cells (hDPSC)/SHED significantly increased bone regeneration [39]. Su et al.’s [40] study utilized 3D-printed scaffolds for bone tissue engineering in stem cell regenerative medicine, aiming to construct functional tissues by mixing stem cells with the scaffolds, mimicking extracellular matrix, bone, and cartilage, and developing disease simulation and stem cell research platforms. It has been shown that the application of biomimetic and biodegradable magnetic scaffolds in oncology and bone tissue engineering provides an appropriate microenvironment for regeneration by delivering cells, growth factors, and drugs to the damaged site [41]. Wu et al. [42] demonstrated that an injectable, antimicrobial calcium phosphate scaffold that promotes bone regeneration, composed of alginate, penicillin, chitosan, and calcium phosphate cement, showed superior strength comparable to cancellous bone. It exerted a prolonged release of penicillin, which successfully stopped the growth of the osteomyelitis-causing bacteria Staphylococcus aureus (S. aureus). Additionally, the scaffold promoted the development and survival of MSCs from human umbilical cords (hUCMSCs). This novel scaffold may improve bone regeneration and treat infections in orthopedic, craniofacial, and dental applications. Its benefits are antibacterial solid action, biocompatibility, injectability, and support for stem cell proliferation. A study conducted by Jiménez et al. [43] on the behavior of human dental pulp MSCs (hDPSCs) on polylactic/polyglycolic acid scaffolds (PLGA) with and without hydroxyapatite showed that the PLGA/HA scaffold significantly induced the differentiation of hDPSCs into osteoblasts, as evidenced by elevated expression of osteogenic markers such as COL-I, RUNX2, ALP, and OPN. Moreover, the two scaffolds had no discernible difference in cell adhesion. Because of its improved performance, the PLGA/HA scaffold has the potential to stimulate hDPSC adhesion, proliferation, and osteogenic differentiation, which in turn promotes bone regeneration in dentistry. Ke et al. [44] investigated feather keratin–montmorillonite nanocomposite hydrogels for bone regrowth. They suggested that these hydrogels stimulate bone repair through the BMP/SMAD signaling pathway, demonstrating their potential in promoting bone tissue regeneration. 

Alksne et al. [45] studied the use of dental pulp stem cell-derived extracellular matrix (ECM) in artificial bone tissue constructs. The DPSC-secreted ECM positively impacted mesenchymal stromal cells, promoting osteogenesis and angiogenic properties. It also recruited endogenous stem cells for bone defects, initiating self-healing. It was found that this DPSC-secreted ECM enhanced the integration of artificial bone constructs and induced successful tissue regeneration. Ismail et al. [46] improved the innovative composite scaffolds for dental applications, specifically bone tissue engineering, using palladium nanoparticles (Pd NPs), a hybrid material containing polyvinyl alcohol and alginate. The scaffolds showed impressive microstructure, shape stability, and oriented lamellar porosity. Pd NPs significantly enhanced their mechanical properties, up to 50 MPa, despite the addition of PVA/Alg. These composite scaffolds exhibit favorable properties for bone regeneration, including biodegradability and osteoconductivity, making them a potential treatment option for bony deficiency. To enhance scaffolds for bone tissue engineering, Valizadeh et al. [47] studied the use of Prunus amygdalus amara (bitter almond) extract in bone tissue engineering scaffolds, revealing that the extract promoted osteogenic differentiation within nanofibrous platforms made from poly-caprolactone and gelatin. The addition of BA to PCL/Gt nanofiber scaffolds improved strain at break, tensile strength, and Young’s modulus. These scaffolds showed increased osteogenic activity and gene expression analysis, aiding in the differentiation of DPSCs into osteoblasts, which enhances their adhesion, proliferation, and osteogenic differentiation. It has been shown that the scaffold’s presence leads hDPSCs to differentiate early in the mineralization medium. In this line, Oliveira et al. [48] demonstrated that human dental pulp stem cells (hDPSCs) exerted typical MSC surface markers and enhanced cell growth when cultured on a polymeric blend scaffold, that was accompanied by increasing levels of osteogenic genes, including Alkaline phosphatase (ALP), type 1 collagen alpha 1 (COL1A1), runt-related transcription factor (Runx-2), and osteocalcin (BGLAP/OCN). A combination of chitosan/gelatin (CS/Gel) scaffolds with DPSCs for orofacial bone reconstruction supported cell proliferation and viability for at least two weeks, induced bone formation, reduced empty spaces, and had higher amounts of osteoid and fully mineralized bone, especially with rhBMP-2 pretreatment [49]. Seeding a calcium phosphate cement (CPC)–metformin scaffold with hPDLSCs led to an increase in osteogenic gene expression and ALP activity, and induced mineral synthesis [50]. In a further study, Lee et al. [51] developed a scaffold for regenerating bone tissue using polydopamine. They attached bone formation peptide-1 to a polycaprolactone 3D scaffold for regenerating bone tissue, which was cultured on human tonsil-derived mesenchymal stem cells. The scaffolds showed osteogenic potential and successfully stimulated vessel and bone regeneration for 8 weeks. It has been shown that tissue-engineered scaffolds can rehabilitate craniofacial bone defects by incorporating hydroxyapatite into chitosan-based hydrogels with increased cell viability and porosity and reducing apatite crystal size [52]. Furthermore, scaffolds that could carry and encapsulate stem cells and bioactive substances would be highly beneficial for dental and craniofacial repairs [53]. An in vivo study conducted by Sun et al. [53] on the bone regeneration ability of an integration of calcium phosphate cement (CPC) scaffold with hPDLSCs and metformin (Met), which were encapsulated within degradable alginate fibers, showed a significant increase in proliferation after alginate fibers were degraded, and hPDLSCs were released. In hPDLSCs, Met delivery significantly increased ALP activity and mineral synthesis. Moreover, CPC+hPDLSCs+ 0.1%Met exhibited the greatest osteogenesis and mineralization compared to other groups without Met. In mandibular bony deficiencies surrounding dental implants, Almansoori et al. [54] investigated the use of a composite scaffold called PCL-TCP, composed of poly (ε) caprolactone and β-tricalcium phosphate, for directed bone regeneration. The MSCs+PRP+PCL-TCP group exhibited notably higher bone density, surface area, and surface-specific area. Additionally, the PCL-TCP scaffold group demonstrated improvements in the ratio of bone-to-implant contact and the development of new bone height, indicating that the combination of PRP and MSCs promotes bone regeneration. Chen et al. [55] developed a bone tissue engineering technique to battle osteomyelitis and post-operative infections in orthopedic, dental, and craniofacial surgery. They embedded human periodontal ligament stem cells (hPDLSCs) and antibiotic ornidazole (ORZ) in a novel injectable calcium phosphate cement (CPC) scaffold. The scaffold inhibited bacterial growth, stimulated osteogenic differentiation, and increased alkaline phosphatase activity. The antibacterial construct was injectable, strong, potent, and biocompatible, offering the potential for treating bone infections and inducing regeneration. It has also been found that the incorporation of ions into the scaffolds inhibits their cytotoxicity, as demonstrated by Zhou et al. [56], who developed silk scaffolds that release a copper peptide, promoting bone regeneration without cytotoxicity. Notwithstanding, copper peptides have therapeutic effects similar to free copper ions but lower toxicity. They promote M2 macrophage polarization and stimulate BMSC growth and cytokine secretion. Liu et al. [57] found that 3D-printed poly-dl-lactin (PDLLA) supports the growth and osteogenic activity of human alveolar bone-derived mesenchymal stem cells (h-ABMSCs), without cytotoxicity, and is compatible with their growth and mineralization. The study of Yang et al. [58] explored autophagy’s role in DPSC migration and regeneration, revealing that SDF-1α is present during pulp angiogenesis in pulpectomized canine teeth. SDF-1α enhances DPSC migration and optimizes focal adhesion formation and stress fiber assembly, while autophagy inhibitors suppress migration. Table 1 presents the studies discussed above at a glance. 

## 3. Exploring Diverse Scaffold Varieties: Unveiling Properties and Efficacy in Fostering Bone Regeneration

Many kinds of scaffolds, each with unique characteristics and functions meant to promote successful tissue restoration, have been used in bone regeneration. Since they are made of inorganic substances such as tricalcium phosphate or hydroxyapatite, ceramic scaffolds are highly biocompatible and closely resemble the mineral makeup of natural bone, which promotes osteoconductivity and tissue integration. Superior bioactivity and biocompatibility are provided by natural polymers like collagen, chitosan, and gelatin, which resemble the extracellular matrix (ECM) of bone [63]. Synthetic polymers offer a flexible environment for cell attachment, growth, and differentiation, but are accompanied by a lack of intrinsic bioactivity compared to natural materials. Composite scaffolds combine materials for improved osteoinductivity, mechanical strength, and bioactivity through combining the benefits of ceramics, natural or synthetic polymers, and a decellularized matrix. Pore-introducing scaffold strategies promote osteointegration and cell infiltration, while growth factor incorporation and coatings enhance the material’s osteoconductive and osteoinductive properties. Further research aims to overcome limitations [64,65]. Magnesium (Mg) is a cell-responsive component used in bone repair materials due to its role in bone metabolism and growth. Mg forms in bone repair scaffolds include powders, oxides, salts (Mg3PO4, MgSiO3, and MgCl2), and ceramic materials. Mg3PO4, soluble in body fluids, produces Mg ions and phosphate ions for bone repair [66,67]. However, the concentration of Mg ions also affected cell proliferation, migration, and osteoblastic differentiation [68]. In this line, Lei et al.’s research [68] utilized 3D printing to develop a scaffold combining Mg3PO4 and polycaprolactone (PCL), showing that PCL scaffolds with 20% Mg3(PO4)2 showed superior osteogenic potential, biocompatibility, and mineralization ability. A study carried out by Chen et al. [69] examined the challenge of promoting new bone formation during oral-guided bone regeneration while preventing fibroblast invasion. Iron oxide nanoparticles (IONPs) were added to a Janus fiber/sponge composite to create an effective barrier membrane. In this composite, a chitosan sponge containing γ-Fe_2_O_3_ was covalently bound to an electrospun layer of poly (lactic-co-glycolic acid)/polycaprolactone (PP). Hemostasis is achieved by the chitosan sponge’s ability to swell and clump blood. IONPs enhanced osteogenesis, while the PP layer prevented epithelial cell invasion and fibroblast penetration. Their study demonstrated that the scaffold effectively promoted bone regeneration in a rat model with calvarial bone injuries. To create multifunctional composite scaffolds that will enhance the treatment of periodontal abnormalities, Zhang et al. [70] studied multifunctional scaffolds with cell barriers and osteogenesis for tissue and bone regeneration. The composite scaffolds were made of various materials, such as nanofibers to stop undesired tissue infiltration, hydrogels, and platelet-rich fibrin to induce bone regeneration, showed exceptional properties and biocompatibility, promoting osteogenic development and potentially treating alveolar bone defects. Nadi et al. [71] synthesized new strontium-doped bredigite (Bre) nanoparticles (called Bresingle bondSr) and incorporated them into three-dimensional scaffolds made of polycaprolactone (PCL) and polylactic acid (PLA). The study found a uniform distribution feature for the functional nanoparticles within the polymer matrix, which increased degradation rates, mechanical strength, and hydroxyapatite crystal formation, supporting human osteoblast viability and proliferation in vitro. The Bresingle bondSr nanoparticles, created through 3D printing, showed promising potential for bone tissue regeneration in rats with critical-sized calvarial defects, demonstrating their suitability for bone regeneration. In another study conducted by Anderson et al. [72], a calcium phosphate-based bioceramic scaffold, termed OsteoinkTM, was developed for treating large and complex alveolar bone defects and designed with two types of calcium phosphate: alginate/x-TCP and hydroxyapatite/x-TCP. The biocompatibility and mechanical characteristics of 3D printed Osteoink scaffolds with alveolar bone marrow stem cells showed maximum compressive strength and superior biocompatibility and fitting clinical defects with high accuracy. This study introduced the Osteoink as a biocompatible material for 3D printing personalized scaffolds. Filippi et al. [63] examined using natural polymeric scaffolds in bone tissue engineering as an alternative to bone autografts to restore damaged bone tissues. Biodegradable matrices (scaffolds) are constructed from natural polymers that are more biocompatible and bioactive than synthetic ones to support cell growth and tissue regeneration. It highlights their combination with other materials and innovative strategies to recreate physiological bone environments. These scaffolds have shown successful results in vitro and in vivo with various cell types and preliminary clinical applications. The study highlights the significant role of natural polymer research in developing effective materials for bone tissue regeneration, potentially having significant clinical applications. Hany et al. [73] studied bone tissue regeneration using a nanofibrous composite scaffold containing polycaprolactone (PCL), alginate (Alg), and hydroxyapatite (HA). The composite showed biocompatibility and suitable properties, with experimental rabbits with mandibular defects showing enhanced bone healing with well-organized and mature bone formation. The scaffold, PCL/Alg/nano-HA, could potentially enhance bone regeneration in mandibular defects. Xu et al. [74] addressed the challenges of bone regeneration in Type II diabetes mellitus. Their study developed 3D-printed bioscaffolds using Sr-containing mesoporous bioactive glass nanoparticles (Sr-MBGNs) in combination with gelatin methacrylate (GelMA). As biomineralization precursors, Sr-MBGNs were embedded in a GelMA-based matrix to release ions (Sr, Ca, and Si) that improve the properties of osteogenesis, angiogenesis, and immunomodulation. The nanocomposites exhibited angiogenic and anti-inflammatory effects through modulation of osteoblast differentiation and the release of bone-related proteins, simulated biomineralization, and activated collagen formation. Their study in patients with Type II diabetes mellitus potentially offers new approaches to bone regeneration by remodeling an unfavorable microenvironment. Three-dimensional biologically active scaffolds (BAs) with poly (lactic-co-glycolic acid) microspheres (PLGA) were studied by Kim et al. [75] with the goal of promoting osteogenic differentiation through encasing 150–250 μm sized nanoparticles with the l-Arginyl-Glycyl-l-Aspartic acid (RGD) sequence (RGD-NPs) and dexamethasone (DEX), a medication that regulates inflammation and differentiation, inside PLGA. Subsequently, the lysine/arginine residues of bone morphogenetic protein 2 (BMP2) formed an ionic bond with the N- and O-sulfates in heparin, immobilizing BMP2 on the surface of microspheres coated with polyethyleneimine (PEI). Their results indicated higher bone differentiation in mice transplanted with NtMPC-containing BAs than in those transplanted with BMSC-containing BAs.

Another biomaterial, magnetic nanoparticles (MNPs), has shown promising results in dental, craniofacial, and orthopedic treatments, offering significant bone repair and regeneration improvements, as Xia et al. [76] explored the use of magnetic nanoparticles (MNPs) in bone tissue engineering, demonstrating advancements in gene modification, cell labeling, targeting, and patterning. Magnetic composite scaffolds and delivery systems for growth factors, medications, and gene delivery can also be produced by MNPs. The new approaches using MNPs, magnetically field-scathed scaffolds, and stem cells improved osteogenic differentiation, angiogenesis, and bone regeneration in comparison to control groups by activating signaling pathways, including MAPK, integrin, BMP, and NF-κB. Wang et al. [77] explored the introduction of tenomodulin, a protein found in the periodontal ligament, using non-viral gene transfection vectors for rat bone tissue regeneration. The study found that the introduction of Tnmd affected hard tissue formation differently depending on the vector used, highlighting the importance of Tnmd in forming dense fibrous tissue. The study found that Tnmd introduction inhibited bone formation in artificial bone defects, depending on the non-viral gene transfection vector used. JetPEI (Tnmd) significantly impacted bone formation, and Tnmd introduction can influence tissue regeneration depending on the transfection vector. Table 2 provides a summary of the studies included in this section.

## 4. The Role of Stem Cells in Enhancing the Bone Regeneration Process

Stem cells are an important tool in promoting bone healing and provide a promising approach to regenerative medicine. MSCs are distinguished by their ability to self-renew and differentiate across several lineages. These cells serve as progenitor cells for osteoblasts, which produce bone, and chondrocytes, which form cartilage and are required for fracture repair [78]. MSCs also release bioactive compounds that regulate cellular activity in different host tissues, which plays a major role in bone-mending processes [79]. MSCs play a vital role in bone healing through interaction with inflammatory stimuli and regenerative cells that proliferate and differentiate into bone tissue [80]. Biomaterial scaffolds, growth factors, and stem cell therapies have demonstrated potential in accelerating bone healing at fracture sites and supporting the regenerative process [81]. To date, numerous stem cell sources with osteogenic and angiogenic potential have been investigated, including the stromal vascular fraction of human adipose tissue [82]. Despite the growing body of in vitro and in vivo research demonstrating MSCs’ ability for bone repair, questions about their exact mechanisms of action and clinical trial effectiveness still need to be answered [83]. Wan et al. [84] presented a study to address the challenges faced by elderly individuals with osteoporosis when using dental implants. The possibility of using adipose-derived stem cells (ADSCs) that have been genetically modified to express high levels of osteoprotegerin (OPG) to treat bone loss in implant dentistry, particularly in situations where there is an estrogen deficiency, was investigated. The study found enhanced proliferation, cell viability, and osteoblast differentiation by introducing Adv-OPG-ADSCs, gene-modified cells, into an osteoporotic rat model created through oophorectomy. Trabecular volume, bone mineral density, and maxillary bone morphology were all markedly enhanced by Adv-OPG-ADSC treatment. Additionally, the therapy suppressed osteoclast generation while promoting osteoblast formation, as indicated by changes in markers and concentrations associated with bone absorption inhibition and formation facilitation. This study established a scientific foundation for utilizing Adv-OPG-ADSCs in treating implant-related osteoporosis, particularly in cases involving estrogen deficiency. Knight and Hankenson [85] discussed the crucial role of MSCs derived from various sources, such as the periosteum, endosteum, and marrow cavity in bone regeneration and fracture healing. Several growth factor signaling pathways influence the differentiation of MSCs, including BMP, Wnt, and Notch. In addition, the study explored the therapeutic potential of manipulating MSCs to enhance bone healing. Niu et al. [86] examined a method for promoting bone regeneration in osteoporosis rats with defective extraction sockets to investigate the impact of BMSCs combined with fibrin glue on the healing of osteoporosis rats’ extraction sockets, as well as the role of estrogen receptors (ERs) in the differentiation of BMSCs and the reconstruction of alveolar bone in osteoporosis rats. The results on rats who underwent bilateral ovariectomies (OVX) to cause osteoporosis and healthy rats showed the osteogenic potential and the expression of the ER mRNA in BMSCs after three months were similar. While BMSCs from osteoporosis rats had lower osteogenic potential and lower ER expression in OVX rats than those from healthy rats, BMSCs combined with FG effectively promoted bone regeneration in them, and the results were comparable to those observed in the sham group. According to the study, BMSCs seeded within FG could be a therapeutic approach for repairing extraction socket defects in osteoporosis. Fujii et al. [87] investigated whether 4-(4-methoxyphenyl) pyrido [40,30:4,5]thieno[2,3-b]pyridine-2-carboxamide (TH), which is derived from helioxanthin, is capable of inducing osteogenic differentiation in hDPSCs. Due to their higher proliferative and clonogenic potential than BMSCs, DPSCs have attracted attention. TH-induced DPSCs are examined in vitro for their ability to induce osteogenesis and in vivo for their ability to stimulate osteogenesis in mouse calvaria defects using cell-sheet technology. Their results showed that TH induces osteogenic differentiation in DPSCs more efficiently than BMP-2 or another condition, indicating significant potential for bone regeneration. The study also demonstrated successful bone regeneration in mice using DPSC sheets treated with TH, demonstrating the practicality of scaffold-free bone regeneration. As a result of their research, TH-induced DPSCs appear to be a promising avenue for bone regenerative medicine, emphasizing the potential for DPSC sheets treated with TH to be used for bone healing and growth factor production without scaffolds or growth factors. Table 3 provides a summary of the studies included in this section.

## 5. General Advantages and Disadvantages of Scaffold Application for Bone Regeneration with Stem Cells in Dentistry

Table 4 provides a balanced view of the pros and cons associated with the use of scaffolds in bone regeneration with stem cells in the field of dentistry. Scaffold-based bone regeneration with stem cells in dentistry offers numerous advantages, including improved biocompatibility, controlled degradation, enhanced effectiveness, versatility in design, mechanical strength, and integration with host tissue. Ongoing innovation and advancements in scaffold technology continue to drive improvements in clinical outcomes and patient care. However, challenges such as immune reactions, inconsistent degradation rates, varying effectiveness, high costs, and regulatory hurdles must be addressed to ensure the safe and effective use of scaffolds in bone regeneration. Customization and scalability remain critical considerations for the widespread adoption of scaffold-based treatments, with modern imaging and 3D printing technologies playing a key role in enabling the creation of patient-specific scaffolds. The regulatory approval process for scaffold-based bone regeneration technologies can be complex and time-consuming, but the potential for faster approval exists if the technology can demonstrate significant clinical benefits. Overall, scaffold-based bone regeneration with stem cells represents a promising and rapidly evolving field of research, with the potential to transform the treatment of bone defects and improve the quality of life for patients in need [35,88,89,90].

## 6. Current Clinical Applications

While still an emerging field, scaffold-based bone regeneration strategies employing stem cells have already found several promising clinical applications within the realm of dentistry:

Periodontal Regeneration: Scaffolds are used to support the regrowth of periodontal tissues damaged by periodontitis. These can include combinations of bone, periodontal ligament, and cementum. Clinical applications often use biodegradable scaffolds seeded with mesenchymal stem cells (MSCs) or growth factors to promote tissue integration and healing.

Alveolar Bone Regeneration: Essential for dental implant stabilization, scaffolds are used to promote bone growth in areas where teeth have been lost. This application is particularly significant in patients with extensive bone loss where traditional implants are not feasible without bone augmentation.

Craniofacial Reconstructions: Scaffolds can help in the reconstruction of bones in patients with congenital facial defects or those resulting from trauma or disease. These applications often require highly customized scaffold designs, which can be achieved with 3D-printing technologies.

Root Canal Therapy: New techniques involve the use of scaffolds to regenerate dental pulp after infections, allowing for more natural tooth preservation compared to traditional root canal treatments [91,92].

## 7. Future Potential

Looking ahead, the future potential of scaffold-based bone regeneration with stem cells in dentistry is vast and holds great promise for enhancing patient care and treatment outcomes. As researchers continue to push the boundaries of biomaterial science and regenerative medicine, several exciting areas of development are emerging that could revolutionize the field.

One area of focus is the development of advanced biomaterials that can actively respond to the biological environment and stimulate specific cellular responses. These “smart” materials may be designed to mimic the natural extracellular matrix more closely, providing a more physiologically relevant environment for cell growth and tissue regeneration. Alternatively, they may be engineered to release growth factors or other bioactive molecules in a controlled manner, enhancing the healing process and improving the overall effectiveness of the scaffold.

Another promising area of research involves the integration of gene editing technologies, such as CRISPR, with scaffold-based therapies. By combining these powerful tools, it may be possible to enhance the regenerative capabilities of stem cells and other progenitor cells, allowing them to produce the desired tissue types more efficiently and effectively. For example, researchers could program cells within the scaffold to produce specific growth factors or signaling molecules that promote bone growth or tissue repair, leading to improved outcomes for patients undergoing bone regeneration procedures.

In addition to these cutting-edge approaches, there is also a growing interest in exploring the use of 3D printing and other additive manufacturing technologies to create custom-designed scaffolds that are tailored to the specific needs of each patient. By combining advanced imaging techniques with computer-aided design and 3D printing, it may be possible to create scaffolds that are perfectly matched to the patient’s anatomy and physiology, providing a more personalized and effective treatment approach.

Overall, the future potential of scaffold-based bone regeneration with stem cells in dentistry is incredibly exciting, with numerous opportunities for innovation and advancement. As researchers continue to explore new materials, technologies, and approaches, we can expect to see significant improvements in the effectiveness, safety, and accessibility of these life-changing treatments, ultimately leading to better outcomes for patients around the world [93,94,95].

## 8. Main Challenges

The application of scaffolds for bone regeneration with stem cells in dentistry is a rapidly evolving field, offering significant therapeutic potential. However, this area also faces various challenges, including regulatory hurdles, safety concerns, and technical limitations (Table 5).

## 9. New Alternative Creative Methods for Regenerating Bone Tissue

The exploration of bone tissue regeneration in dentistry has indeed expanded beyond conventional methods, with the integration of stem cells and 3D scaffolds marking significant advancements. However, there is also a need to broaden the scope of research to include other innovative approaches that do not solely rely on cells, drugs, and scaffolds. Some creative solutions that could be suggested in the paper for regenerating bone tissue are summarized in Table 6.

## 10. Conclusions and Suggestions

Treating bone tissue injuries in the context of oral and dental health is still a difficult task that calls for creative solutions that go beyond accepted methods. Investigating the use of stem cells in conjunction with customized 3D scaffolds has become a viable approach in an effort to overcome the drawbacks of conventional bone grafting. To overcome the drawbacks of traditional bone grafts, tissue engineering approaches that employ scaffolds for stem cell-assisted bone regeneration are essential. This thorough analysis clarifies the critical role scaffolds play in the field of dentistry when it comes to supporting bone regeneration using stem cells. Examining a wide range of research, this review highlights the complex interactions between different kinds of stem cells and scaffolds, emphasizing their combined effect in promoting bone tissue regeneration. It is evident from the reviewed literature that scaffolds play a vital part in promoting successful bone regeneration. These scaffolds function as structural supports, promoting the assimilation and growth of osteogenic cells, specifically mesenchymal stem cells (MSCs), which become essential players in the process of bone formation. The combination of the regenerative capacity of various stem cell lineages with biomaterial scaffolds represents a paradigm shift in bone tissue engineering. The investigation of diverse scaffold types, ranging from ceramics that emulate the composition of natural bone minerals to the biocompatible adaptability of natural and synthetic polymers, has highlighted their pivotal function in fostering osteoinductivity, osteoconductivity, and tissue integration. More investigations into optimized scaffolds, precise interactions with stem cells, and improved vascularization techniques have great potential to improve bone tissue regeneration in dentistry. This study was unable to obtain consistent results from this investigation due to a few constraints, such as the case count. The likelihood grows dramatically with the number of samples. Work that was unethical and immoral in human cases was another drawback. The high cost of using growth agents, which may enhance the amount and quality of regeneration, further limits this study.

The operational suggestions in this study are as follows:

- It is advised to carry out further research on different kinds of oral and dental surgical procedures or other degenerative issues.
- Replacing stem cells with other compounds that promote regeneration when used with scaffolds.

The suggestions in future studies that aim to access a better solution to achieve tissue regeneration are as follows:

- A variety of significant factors, such as growth factors, angiogenic factors inhibitors, insulin-like growth factor (ILGF), vascular endothelial growth factor (VEGF), epidermal growth factor (EGF), and others, can be studied in conjunction with the primary scaffold to influence the enhancement of tissue regeneration or function similarly to stem cells.
- The study group could be different. It is possible to study human cases, but it is time-consuming and legally complex.
- It is advised that a similar study be conducted in orthopedics or another area where bone degeneration results from congenital or pathological conditions, based on the scaffold influence on bone regeneration.
- According to scaffolds’ impact on regeneration enhancement, it could be investigated for use in postponing the aging process.

## Figures and Tables

**Table 1:** Overview of included studies investigating scaffold application for bone regeneration with stem cells.

Author(s)	Type of Scaffold	Type of Stem Cell	Major Points
Kato et al. [59]	Collagen sponge scaffold	Induced pluripotent stem cells derived from peripheral blood cells	They presented a method for purifying osteogenic cells from iPSCs. They have discovered that peripheral blood mononuclear cells (MNCs) can differentiate into osteoblasts, indicating their potential for use in bone regeneration therapy. They provided insight into the potential benefits of MNC-iPSC-derived osteoblasts in the future.
Wu et al. [42]	Antimicrobial calcium phosphate scaffold	Mesenchymal stem cells from human umbilical cords	The scaffold promoted the development and survival of MSCs from human umbilical cords. This novel scaffold may improve bone regeneration and treat infections in orthopedic, craniofacial, and dental applications. Its benefits are antibacterial solid action, biocompatibility, injectability, and support for stem cell proliferation.
Jiménez et al. [43]	Polylactic/polyglycolic acid (PLGA) scaffolds	Human dental pulp mesenchymal stem cells	Examination of the behavior of human dental pulp mesenchymal stem cells on polylactic/polyglycolic acid scaffolds (PLGA) with and without hydroxyapatite (HA). The finding demonstrated that the PLGA/HA scaffold significantly promotes the differentiation of hDPSCs into osteoblasts. The PLGA/HA scaffold has the potential to stimulate hDPSC adhesion, proliferation, and osteogenic differentiation, which in turn promotes bone regeneration in dentistry.
Ke et al. [44]	Keratin–montmorillonite nanocomposite hydrogels	Endogenous stem cells	They showed that the BMP/SMAD signaling pathway is how the hydrogels promote osteogenic development. These hydrogels significantly stimulate bone repair, further supported by in vivo investigations conducted using a rat cranial bone defect model. Their results showed that the feather keratin–montmorillonite nanocomposite hydrogels have excellent potential for therapeutic applications in treating bone diseases.
Alksne et al. [45]	Porous scaffolds	Dental pulp stem cell-derived extracellular matrix (ECM)	The results showed that this DPSC-derived ECM positively impacted mesenchymal stromal cells, enhancing their attachment, migration, proliferation, and promoting spontaneous osteogenesis (bone formation). In the case of bone defects, DPSC-derived ECM attracted endogenous stem cells and started the process of bone tissue self-healing. This DPSC-secreted ECM enhanced the integration of artificial bone constructs and induced successful tissue regeneration.
Ismail et al. [46]	Composite scaffolds	Human dental pulp stem cells	Scanning electron microscopy analysis revealed an impressive microstructure influenced by the concentration of Pd NPs. The finding demonstrated that Pd NPs, in varying concentrations, had a significant impact on the scaffolds’ mechanical properties, enhancing them to levels up to 50 MPa. Scaffolds with Pd nanoparticles can support osteoblast cells, providing stability and a suitable environment for them to grow and form regularly and densely.
Valizadeh et al. [47]	Poly-caprolactone (PCL)/gelatin (Gt) nanofibrous scaffolds with different amounts of bitter almond extract	Dental pulp stem cells	Compared to plain PCL/Gt nanofibers, the scaffolds with BA added had better strain at break, tensile strength, and Young’s modulus. Cell adhesion, spreading, and proliferation tests verified the biocompatibility of BA-infused PCL/Gt scaffolds. Free and BA-loaded scaffolds showed noticeably increased levels of osteogenic activity compared to the control group. According to gene expression analysis, the differentiation of DPSCs into osteoblasts was aided by the upregulation of osteogenic-related genes by free BA and scaffolds loaded with BA.
Al-Qadhi et al. [48]	Nano-bone scaffolds	Bone marrow mesenchymal stem cells (BMSCs) and gingival (GMSCs) mesenchymal stem cells	Their study examined GMSCs for their ability to promote bone regeneration. They concluded that GMSCs may be a viable alternative to BMSCs for the regeneration of bone defects in vivo. A major contribution of this study was the exploration of alternative sources for stem cells in regenerative medicine, particularly in the context of bone regeneration.
Zhou et al. [56]	Silk scaffolds	Bone marrow-derived stem cells (BMSCs)	Copper peptides were found to have therapeutic impacts similar to free copper ions but with lower toxicity when incorporated into scaffolds. The released copper peptide created a microenvironment that stimulated the secretion and proliferation of cytokines by the transplanted BMSCs. They showed that scaffolds seeded with BMSCs result in improved vascularized bone repair when used in vivo to regenerate critical-sized calvarial defects.
Oliveira et al. [60]	Polymeric scaffold	Human dental pulp stem cells	Using these polymeric scaffolds, they evaluated the adhesion, viability, and proliferation of hDPSCs. Their results indicated that hDPSCs displayed typical MSC surface markers and enhanced cell growth when cultured on the polymeric blend scaffold. The ALP activity of the hDPSCs grown in either the mineralization or clonogenic medium exhibited a higher level after 14 days than control cells grown in the clonogenic medium after seeding on the scaffold. An important gain in osteogenic gene expression was observed in cells cultured on the scaffold in a mineralization medium, except BGLAP gene expression.
Zhao et al. [61]	Calcium phosphate cement scaffolds (CPC)	Human periodontal ligament stem cells (HPDLSCS) and human umbilical vein endothelial cells (huvecs)	To promote prevascularization, these constructs were cultured with human platelet lysate (hPL). Prevascularized hPDLSC-hUVEC-CPC-hPL constructs treated in vivo with rats with calvaria defects showed significant bone formation and blood vessel density increases compared to control groups. This study presented an approach for improving cranial bone regeneration, which may find future applications in dental and craniofacial tissue regeneration.
Neves et al. [62]	Chitosan–Xanthan scaffolds with calcium phosphate (Hydroxyapatite and Brushite)	Mesenchymal Stem Cells (MSCs)	This investigation aimed to develop and evaluate polymeric porous scaffolds for regenerative dentistry. This study evaluated the cytotoxicity and biocompatibility of the compounds in vitro and in vivo. The study’s main findings were specific bands in scaffolds related to their components, the effects of calcium phosphates on mechanical characteristics and cell viability, and the influence of MSCs on inflammation. MSCs had a positive effect on reducing the inflammatory response over time.
Bakopoulou et al. [49]	Chitosan/gelatin (CS/Gel) biomimetic scaffold	Dental pulp stem cells (DPSCS)	Both in vitro and in vivo assessments of DPSC behavior were conducted with or without pretreatment with recombinant human BMP-2. In contrast to the control group, these constructs showed increased bone formation, reduced empty spaces, and higher amounts of osteoid and fully mineralized bone. CS/Gel scaffolds combined with DPSCs can facilitate orofacial bone regeneration, especially with rhBMP-2 pretreatment, which has potential implications for future applications.
Zhao et al. [50]	Calcium phosphate cement (CPC)–metformin scaffold	Human periodontal ligament stem cells (HPDLSCS)	Using hPDLSCs obtained from extracted teeth, they prepared CPC scaffolds with and without metformin. Osteogenic differentiation, hPDLSC viability, and mineralization were evaluated over a 14-day culture period, assessing cell attachment, viability, gene expression, alkaline phosphatase activity, and mineralization. Compared to the control group, osteogenic genes were expressed more often, ALP activity was higher, and mineral synthesis was higher.
Lee et al. [51]	Polycaprolactone (PCL) 3D scaffold	Human tonsil-derived mesenchymal stem cells (HTMSCS)	HTMSCs were cultured on scaffolds to assess the scaffold’s biological effects. A rapid differentiation of hTMSCs into osteoblast-like cells demonstrated osteogenic potential. In a rabbit calvarial defect model, these scaffolds successfully stimulated vessel and bone regeneration for 8 weeks. They showed that these osteo-promoting 3D scaffolds in regenerative medicine could potentially provide a safer and more effective bone repair and remodeling method.
Yousefiasl et al. [52]	Chitosan-based hydrogels, modified with alginate and hydroxyapatite	Marrow mesenchymal stem cells	Incorporating hydroxyapatite increased scaffold porosity and pore size, facilitating apatite formation and reducing apatite crystal size. The scaffolds containing 5% hydroxyapatite showed greater cell viability than their control counterparts during biocompatibility tests with rat BMSCs. In their study, printable chitosan-based hydrogel scaffolds with homogeneously distributed hydroxyapatite were developed. They showed that complex craniofacial bone defects may be able to be regenerated with the use of these nanocomposites due to their desirable properties.
Sun et al. [53]	Injectable calcium phosphate cement (CPC) scaffold	Human periodontal ligament stem cells (HPDLSCs)	To integrate the hPDLSCs into the CPC paste, the researchers encapsulated them within degradable alginate fibers. A significant increase in proliferation was observed after alginate fibers were degraded, releasing hPDLSCs. CPC+hPDLSCs+ 0.1% metformin exhibited the greatest osteogenesis and mineralization compared to other groups without metformin. In contrast to the CPC control group, this specific group significantly enhanced bone regeneration nine-fold and increased vascularization three-fold.
Liu et al. [57]	Poly-dl-lactin (PDLLA) scaffolds	Human alveolar bone-derived mesenchymal stem cells (h-ABMSCs)	This study assessed cytotoxicity, proliferation, and osteogenic differentiation of h-ABMSCs on PDLLA using gene expression levels associated with bone formation. h-ABMSCs showed normal proliferation and differentiation when cultured on PDLLA, indicating appropriate characteristics. The PDLLA scaffold did not exhibit cytotoxicity and supported the growth and osteogenic activity of h-ABMSCs. Their results showed that 3D-printed PDLLA is compatible with h-ABMSCs and supports osteogenic differentiation and mineralization of these cells.
Yang et al. [58])	Fibroin scaffold	Dental pulp stem cells (DPSCs)	They aimed to study the involvement of autophagy in the migration of DPSCs and their regeneration mediated by Stromal-Derived Factor 1 alpha (SDF-1α). Their investigation identified the presence of SDF-1α during pulp angiogenesis in pulpectomized canine teeth with complete apical closure. They demonstrated that SDF-1α increased DPSC migration and optimized stress fiber assembly and focal adhesion formation, processes associated with autophagy. Autophagy inhibitors significantly suppressed DPSC migration, while autophagy activators notably increased migration of SDF-1α-stimulated DPSCs.
Almansoori et al. [54]	Composite scaffold, PCL-TCP, composed of poly (ε) caprolactone and β-tricalcium phosphate	Mesenchymal Stem Cells (MSCs)	In their study, various analyses were performed after 12 weeks, including μCT bone morphogenic analysis, fluorochrome bone labeling, and histomorphometric analysis. This group significantly outperformed the PCL-TCP scaffold group in terms of the new bone height formation (NBH) and bone–implant contact ratio. The PCL-TCP scaffold offers improved bone-implant contact and new bone formation in dental implant-associated bone defects, particularly when combined with MSCs and PRP.
Chen et al. [55]	Injectable calcium phosphate cement (CPC) scaffold	Human periodontal ligament stem cells (HPDLSCs)	They aimed to develop a scaffold incorporating ornidazole (ORZ) into a CPC–chitosan matrix and encapsulating hPDLSCs within alginate microbeads, creating a mechanically robust and injectable construct. In addition to exhibiting an inhibition zone on the scaffold, the ORZ-loaded scaffold also inhibited bacterial colonies for up to seven days after loading the scaffold with ORZ. The ORZ-containing scaffold was biocompatible with hPDLSCs. Scaffolds stimulated osteogenic differentiation and bone mineral synthesis in hPDLSCs when combined with osteogenic medium, significantly increasing the activity of alkaline phosphatase and synthesis of bone minerals.

**Table 2:** Summary table of included studies explored the diverse scaffold varieties in bone regeneration.

Author(s)	Type of Scaffold	Their Properties and Effectiveness in Promoting Bone Regeneration
Lei et al. [68]	Combination of magnesium phosphate (Mg3(PO4)2) with polycaprolactone (PCL) scaffold	PCL scaffolds with 20% Mg_3_(PO_4_)_2_ had the best chance of encouraging osteogenic differentiation. In vivo tests on rats with tibial abnormalities and rabbits with maxillofacial deformities verified the scaffold’s ability to regenerate bone. The scaffold, PCL#20MgP, demonstrated exceptional osteogenic potential, biocompatibility, and mineralization ability.
Chen et al. [69]	Janus fiber/sponge composite combining iron oxide nanoparticles (IONPs)	The scaffold showed superior hemostatic ability. IONPs enhanced osteogenesis, while the PP layer prevented epithelial cell invasion and fibroblast penetration. The scaffold effectively promoted bone regeneration in a rat model with calvarial bone injuries.
Zhang et al. [70]	Nanofibers, hydrogels, and platelet-rich fibrin composite scaffolds	The composite scaffolds showed exceptional physical and chemical characteristics, biocompatibility, and the ability to promote osteogenic development in laboratory and animal investigations. The nanofibers created an environment favorable for bone regeneration by preventing connective tissue from penetrating bone flaws. These multifunctional scaffolds have the potential to enhance guided tissue and bone regeneration in the treatment of alveolar bone defects.
Nadi et al. [71]	Nano-biocomposite scaffolds	Compared with scaffolds without nanoparticles, the resulting nanocomposite scaffolds demonstrated increased degradation rates and mechanical strength. They enhanced the formation of hydroxyapatite crystals, an essential component of bone tissue. The scaffolds enriched with Bre-Sr ceramic nanoparticles displayed the most promising potential for regenerating bone tissue.
Anderson et al. [72]	Calcium phosphate-based bio-ceramic scaffold (OsteoInk™)	Compared with hybrid CaP scaffolds, Osteoink scaffolds demonstrated maximum compressive strength and superior biocompatibility. The printed scaffolds demonstrated an excellent degree of accuracy in fitting the clinical defects for which they were intended, with very little morphological deviation from the digital model. Osteoink is a biocompatible material that can be used for 3D printing personalized, clinically acceptable scaffolds tailored explicitly for the reconstruction of alveolar bone.
Filippi et al. [63]	Natural polymeric scaffolds	Biodegradable matrices (scaffolds) are constructed from natural polymers that are more biocompatible and bioactive than synthetic ones to support cell growth and tissue regeneration. It highlights their combination with other materials and innovative strategies to recreate physiological bone environments. These scaffolds have shown successful results in vitro and in vivo with various cell types and preliminary clinical applications.
Hany et al. [73]	Nanofibrous composite scaffold containing polycaprolactone (PCL), alginate (Alg), and hydroxyapatite (HA)	Biocompatibility and suitable physical, chemical, and mechanical properties were demonstrated in the composite scaffold. Enhanced bone healing and mature bone formation were significantly higher than in the control group, as demonstrated by the experimental group, which was well organized. The nanocomposite scaffold [PCL/Alg/nano-HA (nHA)] promised to enhance bone regeneration in mandibular defects.
Xu et al. [74]	3D-printed bioscaffolds using Sr-containing mesoporous bioactive glass nanoparticles (Sr-MBGNS) in combination with gelatin methacrylate (GELMA)	As biomineralization precursors, Sr-MBGNs were embedded in a GelMA-based matrix to release ions (Sr, Ca, and Si) that improve the properties of osteogenesis, angiogenesis, and immunomodulation. The nanocomposites exhibited angiogenic and anti-inflammatory effects through modulation of osteoblast differentiation and the release of bone-related proteins, simulated biomineralization, and activated collagen formation. Their study in patients with Type II diabetes mellitus potentially offers new approaches to bone regeneration by remodeling an unfavorable microenvironment.
Kim et al. [75]	Three-dimensional biologically active scaffolds (bas) comprising poly (lactic-co-glycolic acid) microspheres (PLGA)	In mice receiving transplants of BAs containing BMSCs and NtMPCs, gene expression profiling was conducted using QuantSeq 3 mRNA sequencing. Their results indicated higher bone differentiation in mice transplanted with NtMPC-containing BAs than those transplanted with BMSC-containing BAs.
Xia et al. [76]	Magnetic nanoparticle (MNP) scaffolds	By utilizing MNPs, these magnetic strategies contain targeting, cell labeling, targeting, patterning, and gene modification. MNPs can also create magnetic composite scaffolds and delivery systems for growth factors, drugs, and gene delivery. The new methods combining magnetic nanoparticles, magnetically field-scathed scaffolds, and stem cells improved osteogenic differentiation, angiogenesis, and bone regeneration in comparison to control groups. These mechanisms include activating signaling pathways like MAPK, integrin, BMP, and NF-κB.
Wang et al. [77]	Atelocollagen sponge scaffold	Their results indicated that Tnmd introduction affected hard tissue formation differently depending on the vector used. In vivo, rat calvaria bone defects were treated with Tnmd-introduced scaffolds, and the remaining bone defect volume was assessed. As compared to CaP (Tnmd), JetPEI (Tnmd) significantly impacted bone formation. It has been demonstrated in this study that Tnmd introduction can influence tissue regeneration, depending on the choice of transfection vector when it comes to bone defects.

**Table 3:** Included studies on the role of stem cells in bone regeneration.

Author(s)	Type of Stem Cells	Stem Cell Function in Bone Regeneration
Wan et al. [84]	Adipose-derived stem cells (ADSCs)	Treatment with Adv-OPG-ADSCs significantly improved maxillary bone morphology, trabecular volume, and bone mineral density. Trabecular volume, bone mineral density, and maxillary bone morphology were all markedly enhanced by Adv-OPG-ADSC treatment. This study established a scientific foundation for utilizing Adv-OPG-ADSCs in treating implant-related osteoporosis, particularly in cases involving estrogen deficiency.
Knight and Hankenson [85]	Mesenchymal stem cells (MSCs)	Depending on the type of injury, these MSCs are primarily derived from various sources within the body, such as the periosteum, endosteum, and marrow cavity. This study explored the therapeutic potential of manipulating MSCs to enhance bone healing. To enhance fracture healing, MSCs can be harvested, cultured, and delivered to promote bone regeneration. There is, however, a need to develop agents capable of influencing signal transduction pathways and activating endogenous MSCs further.
Niu et al. [86]	Bone marrow mesenchymal stem cells	Osteogenic potential and the expression of the ER mRNA in osteoporotic and healthy mice BMSCs after three months were similar. BMSCs from osteoporosis rats had lower osteogenic potential and lower ER expression than those from healthy rats. BMSCs combined with FG effectively promoted bone regeneration in OVX rats, and the results were comparable to those observed in the sham group.
Fujii et al. [87]	Human dental pulp stem cells	4-(4-Methoxyphenyl) pyrido[40,30:4,5]thieno[2,3-b]pyridine-2-carboxamide (TH)-induced DPSCs are examined in vitro for their ability to induce osteogenesis and in vivo for their ability to stimulate osteogenesis in mouse calvaria defects using cell-sheet technology. Their results show that TH induces osteogenic differentiation in DPSCs more efficiently than BMP-2 or another condition, indicating significant potential for bone regeneration. The study also demonstrated successful bone regeneration in mice using DPSC sheets treated with TH, demonstrating the practicality of scaffold-free bone regeneration.

**Table 4:** General Advantages and Disadvantages of Scaffold Application for Bone Regeneration with Stem Cells in Dentistry [35,88,89,90].

Aspect	Advantages	Disadvantages
Biocompatibility	- Scaffolds are often made from materials that are compatible with human tissues, minimizing immune reactions.	- Some materials may still trigger minor immune responses or require testing for hypersensitivity.
Degradability	- Designed to degrade at a rate matching new tissue formation, avoiding the need for surgical removal.	- Inconsistent degradation rates can affect the regeneration process and outcomes.
Effectiveness	- Enhances localized stem cell delivery and retention, improving regeneration outcomes.	- Effectiveness can vary based on scaffold architecture and the specific stem cell interactions.
Versatility	- Can be engineered to release growth factors gradually, enhancing healing.	- Complex design requirements can increase costs and production time.
Strength	- Provides mechanical support to the regenerating area, which is crucial in load-bearing regions like the jawbone.	- Some scaffolds may lack sufficient strength until complete tissue integration occurs.
Integration	- Facilitates integration of new tissue with existing bone, promoting stable and enduring regeneration.	- Poor scaffold integration can lead to failure of the regeneration process.
Innovation	- Continuous advancements in material science offer new possibilities for more effective treatments.	- High cost of research and development for new scaffold technologies.
Customization	- Can be customized to fit individual defects using modern imaging and 3D printing technologies.	- Requires precise imaging and manufacturing, adding to the complexity and cost.
Scalability	- Certain scaffold materials and designs are easily scalable for widespread clinical use.	- Scalability can be limited by high material costs and specialized manufacturing processes.
Regulatory	- Potential for faster regulatory approval if proven safe and effective due to high clinical need.	- Regulatory pathways can be lengthy and expensive, delaying market entry.

**Table 5:** Challenges in Scaffold-Based Bone Regeneration with Stem Cells in Dentistry [36,96,97,98].

Category	Challenge	Description
Regulatory Limitations	Approval Processes	- Lengthy and complex approval processes for new biomaterials and stem cell products. - Rigorous testing required for safety, efficacy, and quality control.
	Standardization	- Lack of standardized protocols for scaffold fabrication, characterization, and clinical application. - Need for standardized stem cell procurement, scaffold manufacturing, and application techniques.
	International Regulations	- Differences in regulatory requirements across countries complicate global market access. - Manufacturers must navigate varying legal frameworks and approval pathways.
Safety Issues/Concerns	Biocompatibility and Immunogenicity	- Scaffold materials must be biocompatible to avoid immune reactions. - Potential for immunogenic response increases with material complexity and degradation products.
	Infection Risk	- Scaffolds, especially those seeded with cells, increase infection risk. - Ensuring sterility during production, storage, and implantation is crucial.
	Cell Source and Quality	- Cell source (autologous vs. allogeneic), harvesting, and differentiation protocols affect safety and effectiveness. - Variability in cell quality can lead to inconsistent clinical outcomes.
Technical Limitations	Scaffold Design and Fabrication	- Designing scaffolds that mimic natural bone structure and function is challenging. - Achieving optimal mechanical strength, porosity, and degradation rate is crucial.
	Integration with Existing Tissue	- Ensuring seamless integration with surrounding bone and support for functional, vascularized tissue is essential. - Poor integration can lead to implant failure or suboptimal regeneration.
	Scalability and Cost	- Producing scaffold-based products on a large scale is technically and economically challenging. - Manufacturing consistency, quality control, and cost-effectiveness are crucial.

**Table 6:** Alternative creative methods for regenerating bone tissue [93,99,100,101].

Category	Method	Description
Biophysical Stimuli	Low-Intensity Pulsed Ultrasound (LIPUS)	A non-invasive technology that delivers sound waves to promote cellular activity and angiogenesis, crucial for bone repair and regeneration.
	Electrical Stimulation	Applying electrical currents to enhance osteogenesis by stimulating the proliferation and differentiation of osteoblasts, particularly effective in non-union fractures and large bone defects.
Mechanical Stimulation	Dynamic Loading	Applying mechanical forces to stimulate bone formation through remodeling, using customized mechanical devices or specialized braces.
	Vibration Therapy	Adapting whole-body vibration therapy for localized oral health applications to improve bone density and strength, especially in osteoporotic patients or those with slow healing processes.
Magnetic Field Therapy	Static Magnetic Fields (SMF)	Implementing magnetic devices or materials with inherent magnetic properties to influence bone regeneration by affecting the proliferation and differentiation of osteoblasts.
	Pulsed Electromagnetic Fields (PEMF)	Adapting PEMF for dental applications to accelerate bone regeneration around implants or in areas of bone loss, similar to SMF.
Laser Therapy	Low-Level Laser Therapy (LLLT)	Using specific wavelengths of light to stimulate cellular activity, enhancing bone healing, and reducing inflammation in dental surgeries.
Nutritional Supplements and Diet	Targeted Nutritional Support	Recommending dietary strategies and supplements such as calcium, vitamin D, magnesium, and collagen peptides to support bone health and enhance the efficacy of other regenerative treatments.
	Phytochemicals	Exploring natural compounds with osteo-promotive properties, such as flavonoids found in fruits and vegetables, for their systemic and local effects on bone metabolism.
Gene Therapy	Targeting Bone Growth Factors	Experimentally directing gene therapy to enhance the expression of specific growth factors like BMPs directly at the site of bone injury, bypassing the need for scaffold-based delivery systems.
Biomimetic Materials	Developing Advanced Biomimetic Coatings	Using materials that mimic the natural bone matrix to coat dental implants or integrate into existing scaffold systems, enhancing osteointegration and natural bone growth without the direct use of growth factors or cells.

## Data Availability

Data can be made available on request.
